# The North-Eastern Hospital for Children

**Published:** 1893-05-13

**Authors:** 


### FESTIVAL DINNERS AND MEETINGS.

### THE NORTH-EASTERN HOSPITAL FOR CHILDREN.

THE festival dinner of the North Eastern Hospital for Children took place on the 9th inst. in the Royal Venetian Chamber at the Holborn Restaurant.

The Lord Mayor was in the chair, and amongst the guests present on the occasion were the Duke of Grafton, Lord and Lady F. J. Fitzroy, Mr. Sheriff Renals, Mr. Sheriff Wilkin, Mr. Alderman Pound and Mrs. Pound, Mr. and Mrs. Herring, Mr. and Mrs. Walter Johnson, Captain Kennedy, Mr. Wardlaw, Mr. Knill, Sister Godlee, Dr. Sansom, Mr. H. P. Dean, Dr. Turner, Mr. and Mrs. Maclim, Mr. F. L. A. Gibbs, and many other friends of the hospital.

The LORD MAYOR proposed "The Health of the Queen," which was received with much enthusiasm, and he afterwards gave "The Princes and Princesses," remarking on the willing assistance afforded by them in all works of charity. Referring to a meeting over which he had presided in the afternoon for the Society for the Prevention of Cruelty to Children, the Lord Mayor said he might well repeat a remark that he had made there, it was not charity he asked for the little ones, but rather it was their simple rights that he claimed for them. This speech was well received and warmly applauded, and then the Lord Mayor proceeded to read a letter from Mr. Burdett, who described the little patients as the happiest of hospital children, and urged the importance of raising £20,000 at once to complete the buildings. The Lord Mayor added that from so able a critic the approbation expressed was a very valuable tribute to the management of the hospital. He himself had also visited the wards last Sunday, and was particularly pleased with the kindly tone which pervaded them. He had seen a new child admitted, and was much touched by witnessing the rapidity with which he reconciled himself to his new surroundings and "made a mother of his nurse." There was urgent demand for more room for patients and for nurses, and the land was already secured, but funds were sorely needed to enable the present building to be added to. Adequate hospital accommodation was an imperative necessity in so poor a neighbourhood. The sad cry of "no room" ought to receive prompt attention. The toast of "Prosperity to the North-Eastern Hospital for Children" was most heartily received.

Lord F. J. FITZROY, Chairman of the Hospital Committee, then spoke, and referred to the modest beginning of the little hospital which was started by two ladies.

Sheriff RENALS made a speech; and Mr. GODLEE, the Treasurer, told of the financial anxieties of the committee, and also of the intense interest in the well-being of the hospital which was evinced by all connected with it. He highly commended the earnest work of the matron and her staff, and spoke appreciatively of the exceptionally talented medical staff. By all the speakers the needs of the children were most eloquently urged, and "help for those who cannot help themselves" was asked. Mr. WALKER JOHNSON related with much humour the experiences of stewards who collected for charities. The energetic SECRETARY, Mr. Alfred Nixon, who had been far too busy during the evening in attending to other people's comfort to have any time to spare for his own dinner, read a list of subscriptions and donations received amounting to about £1,100.

In thanking the Lord Mayor for so kindly presiding, the Duke of GRAFTON expressed regret for the general depression which had such a disastrous effect on all charities at present, he thought the small sums which people hesitated to offer became of substantial benefit when collected systematically.

The Lord Mayor's health was drunk with acclamation, and in acknowledgment his Lordship remarked that he was much touched by the many kind expressions used towards him. For his part he delighted to honour children at all times. The spirit of charity must be encouraged not discouraged. Other speeches followed, great satisfaction being expressed at the presence of so many ladies. Good results should follow this exhibition of general interest in a most deserving institution, which was very fully described, as our readers will remember, in THE HOSPITAL of May 6th.

